# SphK2 over-expression promotes osteosarcoma cell growth

**DOI:** 10.18632/oncotarget.22314

**Published:** 2017-11-06

**Authors:** Dawei Xu, Hao Zhu, Chengniu Wang, Wei Zhao, Genxiang Liu, Guofeng Bao, Daoran Cui, Jianbo Fan, Fei Wang, Huricha Jin, Zhiming Cui

**Affiliations:** ^1^ Department of Orthopaedics, The Second Affiliated Hospital of Nantong University, Nantong, China; ^2^ Department of Orthopaedics, The Fourth Affiliated Hospital of Nantong University, Yancheng, China; ^3^ Basic Medical Research Centre, Medical College, Nantong University, Nantong, China

**Keywords:** osteosarcoma, sphk2, microRNA-19a-3p, oncotarget

## Abstract

It is needed to explore novel biological markers for early diagnosis and treatment of human osteosarcoma. Sphingosine kinase 2 (SphK2) expression and potential functions in osteosarcoma were studied. We demonstrate that SphK2 is over-expressed in multiple human osteosarcoma tissues and established human osteosarcoma cell lines. Silence of SphK2 by targeted-shRNAs inhibited osteosarcoma cell growth, and induced cell apoptosis. On the other hand, exogenous over-expression of SphK2 could further promote osteosarcoma cell growth. Notably, *microRNA-19a-3p* (“*miR-19a-3p*”) targets the 3′ UTR (untranslated region) of *SphK2 mRNA*. Remarkably, forced-expression of *miR-19a-3p* silenced SphK2 and inhibited osteosarcoma cell growth. *In vivo*, SphK2 silence, by targeted-shRNA or *miR-19a-3p*, inhibited U2OS tumor growth in nude mice. These results suggest that SphK2 could be a novel and key oncotarget protein for OS cell progression.

## INTRODUCTION

Over the past decades, the prognosis of osteosarcoma (OS) has been significantly improved [1–6]. Significant advances in OS early diagnosis and treatments, including surgery, radiotherapy, and chemotherapy, have been achieved [1, 5, 7–9]. Yet, the overall survival has reached a platform [10–13]. Further, the prognosis of patients with recurrent and metastatic OS is poor, but the incidence of OS is rising at a rate of 1.4% per year [11–13]. OS has become one leading cause of cancer-related mortalities among children and teenagers [10–13]. Therefore, it is needed to identify reliable oncotarget proteins for early diagnosis and treatment of OS [5, 14, 15].

The cellular sphingolipid signaling is involved in tumorigenesis and cancer progression [16–19]. Production of pro-apoptotic sphingolipids, *i.e.* ceramide, will potently inhibit cancer cells. Reversely, anti-apoptotic sphingolipid sphingosine-1-phosphate (S1P) will promote cancer cell survival and growth [20–22]. The balance of these sphingolipids is tightly controlled by sphingosine kinase (SphK) [20]. Over-activation or upregulation of SphK would lead to increased conversion of ceramide to S1P, causing aberrant cell growth [20, 23]. At least two SphKs have been identified, including the well-studied SphK1 and the less-known SphK2 [20]. Recent studies have proposed that SphK2 is over-expressed in many cancer cells [24–26]. Its expression and potential functions in human OS were tested in this study.

## RESULTS

### Over-expression of SphK2 in human OS tissues and OS cells

**Figure 1:**
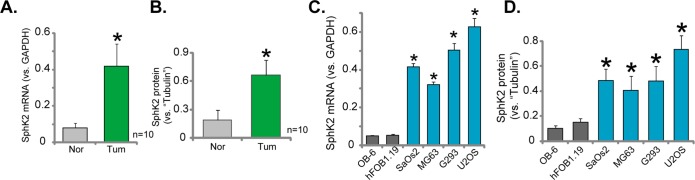
Over-expression of SphK2 in human OS tissues and OS cells mRNA **(A** and **C)** and protein **(B** and **D)** expression of SphK2 in OS tissues (“Tum”) and the surrounding normal bone tissues (“Nor”), as well as in human OS cells (MG63, SaOs2, G293 and U2OS lines) and human osteoblastic cells (OB-6 and hFOB1.19 lines), were shown. Relative SphK2 protein expression (vs. Tubulin, B and D) was quantified. ^*^p<0.05 vs. “Nor” tissues (A-B). ^*^p<0.05 vs. OB-6 cells (C-D).

First, we tested the expression of SphK2 in human OS tissues. A total of ten OS tissues (“Tum”) and their surrounding normal bone tissues (“Nor”) were examined. Quantitatively real-time PCR (“qRT-PCR”) assay results showed that *SphK2 mRNA* expression in the OS tissues was significantly higher than that in the bone tissues (Figure 1A). *SphK2 mRNA* in tumor tissues was about 4-5 times higher than the normal tissues (Figure 1A). Meanwhile, SphK2 protein was also upregulated in OS tissues (the quantified results in Figure 1B), and its expression was relative low in normal tissues (Figure 1B).

SphK2 expression in human OS cells was also tested. Four well-established human OS cell lines, MG63, SaOs2, G293 and U2OS, as well as two lines of human osteoblastic cells (OB-6 and hFOB1.19) were tested. qRT-PCR assay results in Figure 1C confirmed that *SphK2 mRNA* level in the OS cells was significantly higher than that in the osteoblastic cells. Consequently, SphK2 protein expression was elevated in OS cells (Data were quantified, Figure 1D). Among all the tested OS cell lines, SphK2 expression was highest in U2OS cells (Figure 1C and 1D), this cell line was selected for further studies. Together, these results clearly demonstrate that SphK2 is over-expressed in human OS tissues and OS cells.

### SphK2 knockdown inhibits OS cell growth

**Figure 2:**
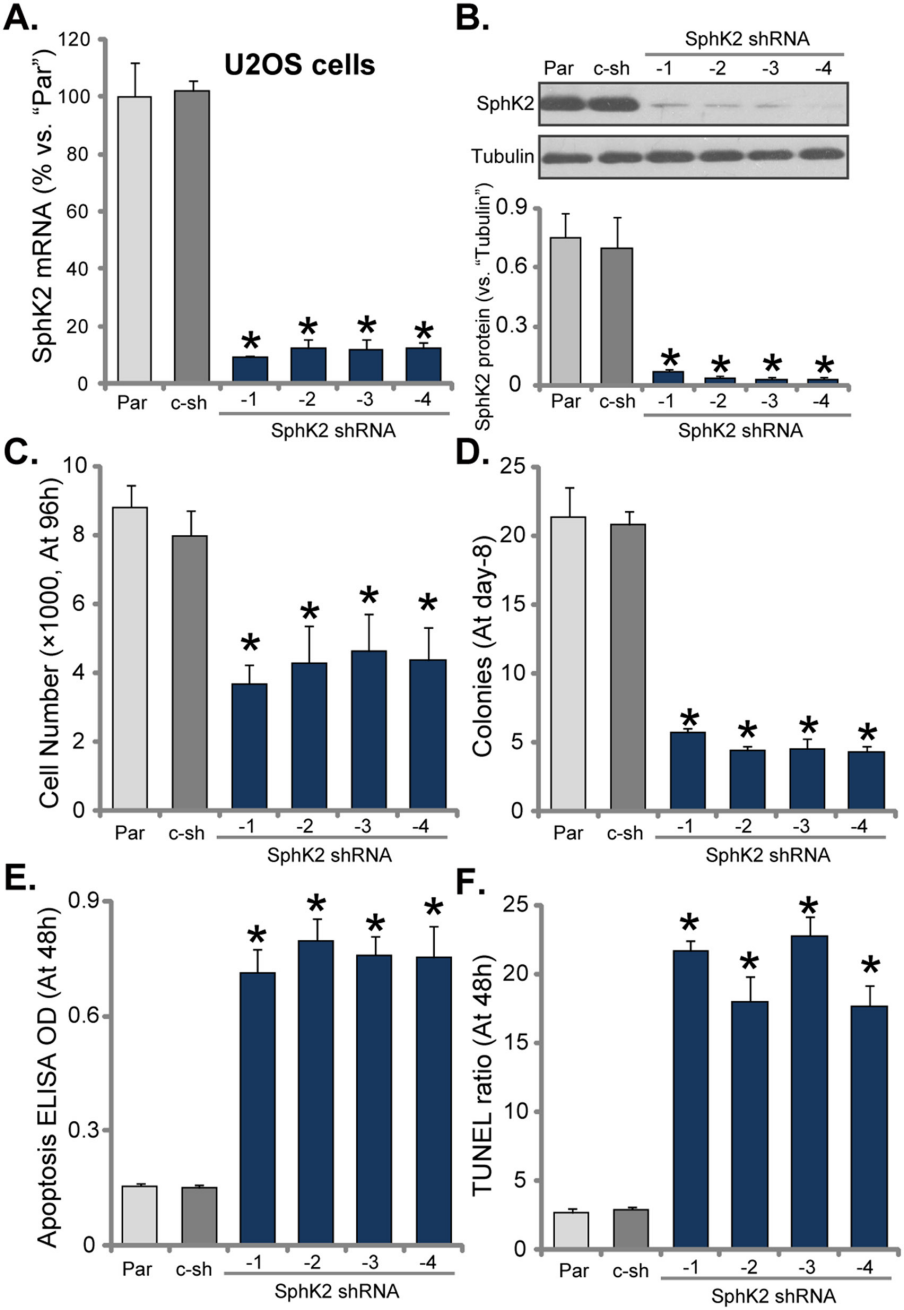
SphK2 knockdown inhibits OS cell growth mRNA **(A)** and protein **(B)** expression of SphK2 in stable U2OS cells, expressing SphK2 shRNA (“-1 to -4”) or scramble non-sense control shRNA (“c-sh”), as well as in parental control U2OS cells (“Par”), were shown. Cells were also subjected to cell counting assay **(C)** and colony formation assay **(D)** to test cell growth; Cell apoptosis was examined by DNA apoptosis ELISA OD **(E)** and TUNEL assay **(F)**. For each assay, exact same amount of viable cells of different genetic modification were plated initially (Same for all Figures). Data were shown as mean (n=5) ± standard deviation (SD). ^*^p<0.05 vs. “Par” cells. Experiments in this figure were repeated three times, and similar results were obtained.

To study the potential function of SphK2 in OS cells, we utilized shRNA method to knockdown SphK2 in U2OS cells. As discussed, a total of eight (8) distinct lentiviral shRNAs, against non-overlapping sequence of human SphK2, were designed. Of which, four of them efficiently silenced SphK2 in U2OS cells, these four shRNAs were named as SphK2 shRNA (“-1/−4”). As demonstrated, *SphK2 mRNA* level was significantly downregulated in the stable U2OS cells expressing the SphK2 shRNAs (Figure 2A). Consequently, SphK2 protein was also silenced (Figure 2B, data was quantified). Remarkably, the growth of U2OS cells was significantly inhibited after SphK2 knockdown (Figure 2C and 2D), as the number of viable cells (at 96 hour, Figure 2C) and colonies (at Day-8, Figure 2D) were both significantly decreased after expressing SphK2 shRNAs. Notably, scramble non-sense control shRNA (“c-sh”) showed no effect on SphK2 expression (Figure 2A-2B) nor U2OS cell growth (Figure 2C-2D). Thus, SphK2 knockdown by targeted shRNAs inhibited U2OS cell growth.

Apoptosis induction could be a main reason of growth inhibition in OS cells. Next, Histone DNA apoptosis ELISA assay and TUNEL staining assay were performed to test the potential effect of SphK2 shRNA on cell apoptosis. DNA denaturation occurs in the apoptotic cells, which binds to histone. Thus, testing histone-bound DNA could be an efficient way to examine cell apoptosis [27]. TUNEL nuclei staining assay, which tests DNA fragmentation in nuclei, is a well-established apoptosis assay [28]. As demonstrated, Histone DNA apoptosis ELISA optic density (OD, Figure 2E) and the TUNEL-nuclei percentage (Figure 2F) were both increased after expressing SphK2 shRNA in U2OS cells, suggesting that SphK2 shRNA induced apoptosis in U2OS cells. The control shRNA was again in-effective (Figure 2E and 2F). Similar results were also obtained in MG63 OS cells, where knockdown of SphK2 by targeted shRNA (“-1”) (Supplementary Figure 1A) inhibited cell growth (Supplementary Figure 1B), but induced cell apoptosis (Supplementary Figure 1C).

### Exogenous over-expression of SphK2 promotes OS cell growth

**Figure 3:**
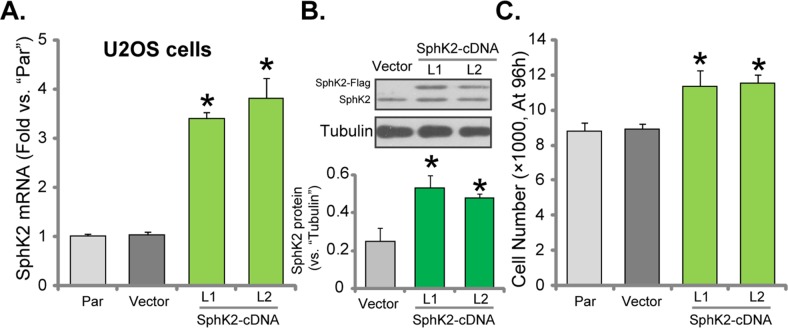
Exogenous over-expression of SphK2 promotes OS cell growth mRNA **(A)** and protein **(B)** expression of SphK2 in stable U2OS cells expressing SphK2 cDNA (two lines, “SphK2-cDNA-L1/L2”) or the empty vector (pSuper-EGFP-puro-Flag), as well as in parental control cells (“Par”), were shown; Cells were also subjected to cell counting assay **(C)** to test cell growth. Data were shown as mean (n=5) ± standard deviation (SD). ^*^p<0.05 vs. “Par” cells. Experiments in this figure were repeated three times, and similar results were obtained.

To further confirm the potential function of SphK2 in promoting OS cell growth, the SphK2-expression vector (Flag-tagged, see Methods) was established. The construct was transfected to U2OS cells. Via puromycin selection, two stable U2OS cell lines expressing the construct were established, which were named as “SphK2-cDNA-L1/L2”. qRT-PCR assay results in Figure 3A demonstrated the significant upregulation of *SphK2 mRNA* in the stable cells with the construct. Furthermore, Western blotting assay results confirmed the expression of exogenous SphK2 (“Flag-tagged”) in the stable cells (Figure 3B). Significantly, U2OS cell growth, tested by the simple cell counting assay (Figure 3C), was facilitated after SphK2 over-expression. Similar results were also obtained in MG63 cells, where over-expression of SphK2 (Supplementary Figure 1D) facilitated cancer cell growth (Supplementary Figure 1E). The empty vector (pSuper-puro-Flag) had no significant effect on SphK2 expression (Figure 3A and 3B) and cell growth (Figure 3C). Together, these evidences conclude that exogenous over-expression of SphK2 could further promote OS cell growth.

### Expression of microRNA-19a silences SphK2 and inhibits OS cell growth

**Figure 4:**
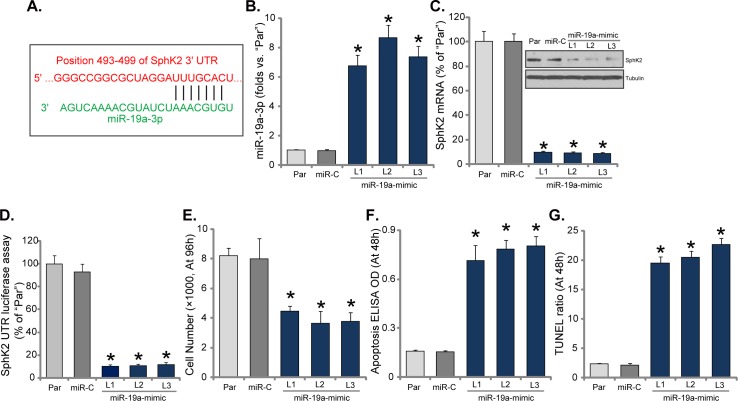
Expression of microRNA-19a silences SphK2 and inhibits OS cell growth miR-19a-3p putatively targets the 3′ UTR of *SphK2 mRNA* **(A)**. Expression of *miR-19a-3p* **(B)** and SphK2 (protein and mRNA, **C**), as well as the relative *SphK2 mRNA* 3′-UTR luciferase activity **(D)** in the stable USO2 cells with miR-19a-mimic (three lines, “L1-L3”) or miR-control (“miR-C”), and in the parental control cells (“Par”) were shown; Cells were also subjected to the cell counting assay **(E)** and the listed apoptosis assays **(F** and **G)**. Data were shown as mean (n=5) ± standard deviation (SD). ^*^p<0.05 vs. “Par” cells. Experiments in this figure were repeated three times, and similar results were obtained.

Above results have shown that SphK2 is over-expressed in OS cells, which is important for cell growth. The potential mechanism of SphK2 upregulation in OS cells was tested. Here we focused the possible mechanism of microRNA. Via search the “TargetScan” database, we discovered one potential anti-SphK2 microRNA: *microRNA-19a-3p* (“*miR-19a-3p*”). As demonstrated, *miR-19a-3p* putatively targets the 3′ UTR (untranslated region) of *SphK2 mRNA* (Figure 4A). Next, miR-19a-mimic oligonucleotides were transfected to U2OS cells, and stable cells (three lines, “L1-L3”) expressing *miR-19a* were established. qRT-PCR assay results in Figure 4B confirmed that *miR-19a-3p* level was significantly increased in all three stable lines with miR-19a-mimic. Remarkably, *SphK2 mRNA* and protein were both significantly downregulated after expressing *miR-19a* (Figure 4C). Thus, forced-expression of *miR-19a-3p* downregulated SphK2 in U2OS cells. Additionally, the *SphK2 mRNA* 3′-UTR luciferase activity was also largely inhibited in *miR-19a*-expressing U2OS cells, indicating that SphK2 should be the direct target of *miR-19a*.

Further studies showed that U2OS cell growth, tested by cell counting assay, was also inhibited after stably expressing *miR-19a-3p* (Figure 4E). On the other hand, apoptosis level was increased in the stable cells with *miR-19a-3p* (Figure 4F and 4G). In the *miR-19a*-expressing cells, Histone DNA ELISA OD (Figure 4F) and TUNEL percentage (Figure 4G) were both increased. Expression of miR-control (“miR-C”), expectably, didn't change SphK2 expression (Figure 4C) nor cell growth (Figure 4E). Similar results were also observed in the MG-63 cells. Introduction of miR-19a-mimic significantly increased *miR-19a-3p* expression (Supplementary Figure 2A), which downregulated SphK2 protein and mRNA (Supplementary Figure 2B), causing growth inhibition (Supplementary Figure 2C) and cell apoptosis ((Supplementary Figure 2D). Thus, expression of *miR-19a-3p* downregulates SphK2 and inhibits OS cell growth.

### Downregulation of miR-19a-3p in human OS tissues and cells

**Figure 5:**
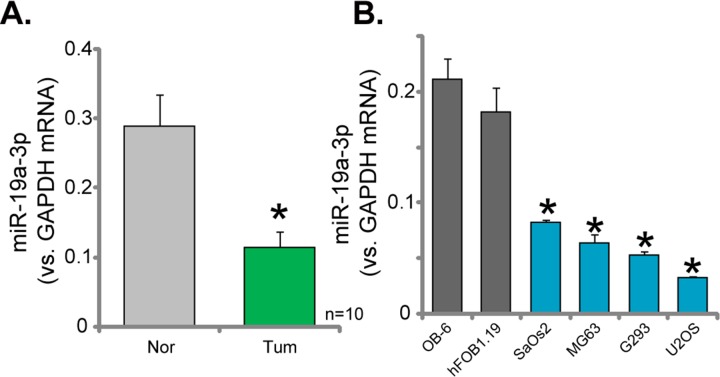
Downregulation of *miR-19a-3p* in human OS tissues and cells Relative*miR-19a-3p* expression (vs. *GAPDH mRNA*) in OS tissues (“Tum”) and surrounding normal bone tissues (“Nor”) **(A)**, as well as in human OS cells (MG63, SaOs2, G293 and U2OS lines) and human osteoblastic cells (OB-6 and hFOB1.19 lines) **(B)**, were shown. Data were shown as mean (n=5) ± standard deviation (SD). ^*^p<0.05 vs. “Nor” tissues (A). ^*^p<0.05 vs. OB-6 cells (B).

Expression of *miR-19a-3p* in the above-mentioned human OS tissues (See Figure 1) was also tested. As demonstrated in Figure 5A, *miR-19a-3p* level was significantly lower in the OS tissues, as compared to the surrounding normal tissues. Further, *miR-19a-3p* level in all four lines of human OS cells (MG63, SaOs2, G293 and U2OS) was also lower than that in the osteoblastic cells (OB-6 and hFOB1.19 lines) (Figure 5B). These results confirmed *miR-19a-3p* downregulation in human OS tissues and cells.

### Silence of SphK2 inhibits U2OS tumor growth in nude mice

**Figure 6:**
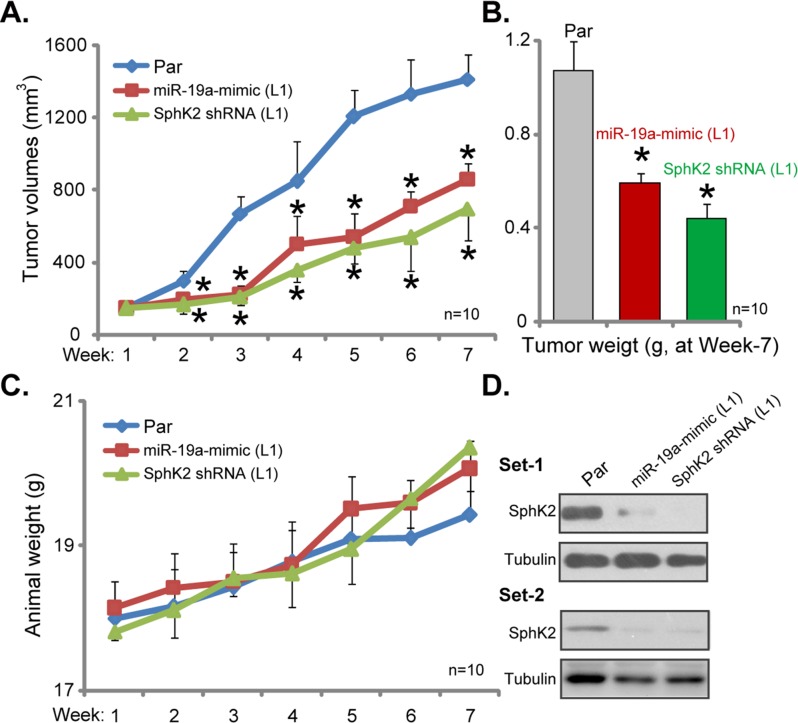
Silence of SphK2 inhibits U2OS tumor growth in nude mice Parental U2OS cells (“Par”), as well as cells stably expressing SphK2-shRNA (“L1”) or *miR-19a-3p* (“L1”), were injected *s.c.* to the nude mice; Estimated tumor volume **(A)** and mice body weight (deducting tumor weight, **C**) were recorded weekly for total six weeks. At the end of experiment (week-7), tumors of the each group were isolated and weighted **(B)**, expression of listed proteins in tumor lysates was shown (Two sets, **D**). ^*^p<0.05 vs. “Par” tumors.

At last, the potential effect of SphK2 on OS cell growth *in vivo* was tested. Parental U2OS cells, as well as cells stably expressing SphK2-shRNA or *miR-19a-3p*, were injected *s.c.* to the nude mice. Xenografted U2OS tumors were established within 2-3 weeks. Weekly tumor growth was recorded. Estimated tumor growth curve results in Figure 6A demonstrated that U2OS tumors expressing SphK2-shRNA or *miR-19a-3p* grew significantly slower than the control tumors (from parental cells). The volumes of tumor expressing SphK2-shRNA or *miR-19a-3p* were much lower than the control tumors (Figure 6A). At the end of experiment (week-7), tumors of each group were isolated and weighted. Results in Figure 6B confirmed that SphK2-shRNA- or *miR-19a-3p*-expressing tumors were dramatically lighter than the control tumors. Notably, mice body weights were not significantly different between the groups (Figure 6C). When analyzing tumor tissue lysates, we showed that SphK2 expression was indeed downregulated in tumors expressing SphK2-shRNA or *miR-19a-3p* (Figure 6D, two sets). These results suggest that knockdown of SphK2, by targeted-shRNA or *miR-19a-3p*, inhibited U2OS tumor growth in nude mice.

## DISCUSSIONS

The oncogenic/pro-cancerous function of SphK1 has been well-established [23, 29, 30]. On the other hand, the potential function of SphK2 in human cancers has not been extensively studied until recently [24, 26, 31, 32]. Although early studies have proposed a possible pro-apoptotic/anti-cancer function by of SphK2 [33], emerging recent evidences have confirmed that SphK2, like SphK1, is also oncogenic [24, 26, 31, 32]. It is known that SphK2 could also enzymatically produce S1P from sphingosine, although its efficiency is less potent than SphK1 [26, 34]. Meanwhile, SphK2 activation can induce G1/S transition and promote cell proliferation [26, 34]. SphK2 is also shown to be important for oncogenic AKT activation and ERK2 expression. On the other hand, SphK2 knockdown resulted in AKT inactivation and ERK2 downregulation, causing proliferation and migration suppression [34]. SphK2 is important for MYC expression to promote acute lymphoblastic leukemia progression [35]. Over-expression of SphK2 is associated with multi-drug resistance [26, 36]. ABC294640, a selective SphK2 inhibitor, has anti-proliferative and antitumor activity in a variety of cellular and animal models [31, 37, 38]. The SphK2 inhibitor is now under phase II clinical trials [31, 32, 38–42]. Thus, targeting SphK2 should have therapeutic potential for treating cancer [24, 26, 31, 35, 43].

The preclinical evidences of this study suggested that SphK2 is possibly a rational therapeutic target for human OS. First, SphK2 expression is upregulated in multiple human OS tissues, as well as in OS cell lines. Its level was however quite low in normal bone tissues and in human osteoblastic cells. Second, stable knockdown of SphK2 by targeted-shRNAs largely inhibited U2OS cell growth, and induced cell apoptosis. On the other hand, exogenous over-expression of SphK2 could further promote U2OS cell growth. Third, forced-expression of *miR-19a-3p*, the potential anti-SphK2 miRNA, silenced SphK2 and inhibited U2OS cell growth. Fourth, SphK2 silence, by targeted-shRNA or *miR-19a-3p*, dramatically inhibited U2OS tumor growth in nude mice. Fifth, our preliminary studies have demonstrated that ABC294640, the SphK2 specific inhibitor [31, 38, 42], inhibited OS cell growth *in vitro* and *in vivo*. Thus, SphK2 could be a key oncotarget protein for human OS.

miRNA binds to the 3′ UTR of the targeted mRNA, causing translational inhibition and/or mRNA degradation [44–46]. It has been aware that dysregulation of miRNA participates in a number of cancerous behaviors, including cell survival, growth and cell cycle progression as well as apoptosis and migration, metabolism [47–50]. Dysregulation of miRNA is also the characteristic marker of human OS [51–53]. One novel finding of this study is that *miR-19a-3p* could be the specific SphK2-targeting miRNA. *miR-19a-3p* putatively targets the 3′UTR of *SphK2 mRNA*. Significantly, forced-expression of *miR-19a-3p* downregulated SphK2 and efficiently inhibited U2OS cell growth. More importantly, *miR-19a-3p* level was decreased in the OS tissues/cells, corresponding to SphK2 upregulation. It is therefore possible that *miR-19a-3p* downregulation is the cause of SphK2 upregulation in human OS cells, although this hypothesis warrants further investigations.

It should be noted that the same miRNA (*miR-19a-3p* in our study) might have other target proteins and exert different and sometimes contradictory functions in cancer progression [54]. Furthermore, expression a single miRNA, *miR-19a* for example, could exhibit reverse activities under different contexts [55–57]. Therefore, it will be interesting to further understand the functional complexity of *miR-19a*, and to verify it as a potential anti-OS miRNA.

## MATERIALS AND METHODS

### Reagents

Puromycin was purchased from Sigma Aldrich (St. Louis, MO). All the antibodies were purchased from Cell Signaling (Beverly, MA).

### Human tissue specimens

The human OS tissues along with the surrounding normal bone tissues were obtained at the time of surgery, and were separated carefully under microscope. Tissues were washed with PBS plus antibiotics, and were minced into small pieces, which were then mechanically dissociated and lysed by the tissue lysis buffer (Biyuntian, Nantong China). Tissue lysates were stored in liquid nitrogen for further analysis. A total of ten OS patients were enrolled, which were provided with written-informed consent. The protocol was according to the principles expressed in the Declaration of Helsinki, and was approved by the Ethic Board of Nantong University.

### Cell culture

The human OS cell lines, including MG63, SaOs2, G293 and U2OS, as well as two lines of human osteoblastic cells (OB-6 and hFOB1.19) [49, 58] were obtained from the Cell Bank of Shanghai Institute of Biological Science (Shanghai, China). All the cell lines were cultured in DMEM/F12 supplemented with 8-10% FBS, and were maintained at 37°C in the presence of 5% CO_2_.

### Clonogenic assay of cell growth

Cells were plated at 500 cells/well onto the six-well plate and were incubated for 8 days, which were then fixed with methanol-acetic acid solution and stained with crystal violet. The number of colonies was manually counted under the light microscopy.

### Fragmented DNA detection by ELISA

Nucleosomal DNA fragmentation, the characteristic marker of cell apoptosis, was tested via measuring Histone-bound DNA using a specific two-site ELISA kit (Roche, Shanghai, China), including an anti-histone primary antibody and a secondary anti-DNA antibody. ELISA OD at 450 nm was recorded as the quantitative measurement of cell apoptosis [59].

### TUNEL assay of apoptosis

U2OS cells with distinct genetic treatment were subjected to the TUNEL dye assay. Positive TUNEL staining is a well-known indicator of cell apoptosis. At least 200 cells of five random views of same condition were analyzed. TUNEL ratio (vs total cell nuclei, Hoechst staining) was recorded.

### Western blotting assay

Equivalent amount of lysate proteins (30 μg per lane) were separated by 8-10% of SDS-PAGE gels, and were transferred to PVDF membranes (Millipore, Shanghai, China). The blots were then blocked, and were probed with the indicated primary and secondary antibodies. The protein signals were visualized under the ECL detection kit. β-Tubulin (“Tubulin”) was always tested as the loading control. Quantification of the signal was performed through the Image J software [60], analyzing total gray of each band.

### SphK2 shRNA

A total of eight distinct lentiviral shRNAs, against non-overlapping sequence of human SphK2, were designed, synthesized and verified by Genepharm Company (Shanghai, China). The lentiviral shRNA (10 μL/mL, per well) was added to cultured U2OS/MG-63 cells for 24 hours. Stable cells were selected by puromycin (2.5 μg/mL, Sigma) for another 6-8 days. SphK2 expression in the stable cells was tested by Western blotting assay. Control cells were infected with lentiviral scramble control shRNA (Santa Cruz Biotech).

### SphK2 over-expression

The full length human *SphK2 cDNA*, provided by Genepharm Company, was inserted into the pSuper-puro-EGFP-Flag vector (Addgene, Shanghai, China) to establish SphK2 expression construct. The latter was transfected to U2OS/MG-63 cells via Lipofectamine 2000 regent [61]. Stable cells were selected by puromycin (2.5 μg/mL, Sigma) for another 6-8 days. Both endogenous and exogenous (Flag-tagged) SphK2 expression in the stable cells was tested by Western blotting assay.

### Quantitative RT-PCR

Total cellular RNA was extracted through the Trizol reagents (Invitrogen, Shanghai, China), and cDNA was synthesized from 0.5 μg mRNA using High Capacity cDNA Reverse Transcription Kit according to the manufacturer's instruction. Quantitative real-time PCR (“qRT-PCR”) assay by Power SYBR Green RT-PCR Reagents Kit was performed via the ABI7500 system. The primers for *SphK2 mRNA* were 5′-TTCTATTGGTCAATCCCTTTGG-3′ and 5′-AGCCCGTTCAGCACCTCA-3′. The primers GAPDH *mRNA* were described early [62]. The 2-^ΔΔCt^ was calculated to yield *SphK2 mRNA* fold expression (relative to GAPDH). miR-19a-3p expression was tested via the TaqMan microRNA assay (Applied Biosystems, Shanghai, China), from 5 ng of total RNA [63].

### miR-19a transfection

U2OS/MG-63 cells were transfected with 20 nM of miR-19a mimic oligonucleotides (Ambion, Shanghai, China) by Lipofectamine 2000 (Invitrogen). After two days, cells were split and were transfected with miR-19a mimic again. This process was repeated for eight rounds for a total of 16 days when necessary, until stable cell lines were established. Expression of miR-19a-3p in the stable OS cells was tested by the qRT-PCR assay. The Ambion Pre-miRNA Precursor Negative Control (“miR-C”) was introduced to OS cells as the control cells.

### *SphK2 mRNA* 3′-UTR luciferase activity assay

The reporter vector with the 3′-UTR of *SphK2* carrying a putative miR-19a-3p binding site (Position 493-499) was designed, constructed, sequence-verified by Genepharm (Shanghai, China). The complementary oligonucleotides for the selected region were hybridized to form double-stranded DNA and inserted into pmIR-ReporterTM firefly luciferase vector (Genepharm, Shanghai, China). The construct was transfected together with the *miR-19a* mimic to U2OS cells. Cells were subjected to the luciferase assay using the commercial available kit (Promega, Shanghai, China). The luciferase of β-galactosidase was utilized as an internal control.

### U2OS xenograft assay

All experimental protocols involving the nude mice were approved by Nantong University's Ethics Board. Female nude mice (6-8 weeks age, 17.5-18.8 g weight) were subcutaneously (s.c.) inoculated with 5 × 10^6^ U2OS cells (in 0.2 mL DMEM/10% FBS), with/out SphK2-shRNA or miR-19a-3p, into the right flanks. When U2OS xenograft volumes reached about 150 mm^3^, the recordings were started. The xenografted tumor volumes along with mice body weights were recorded every week for a total of six weeks [64]. At the end of experiment (week-7), tumors of each group were isolated and weighted. Tumor tissues were subjected to Western blotting assay of indicated signaling proteins.

### Statistics

Data were expressed as the mean ± SD (Standard Deviation). Comparisons between groups were performed via one-way ANOVA (SPSS 18.0) followed by Bonferroni post hoc test. *p* values < 0.05 were considered statistically significant.

## CONCLUSIONS

In summary, SphK2 over-expression promotes OS cell growth. SphK2 could be a novel and key oncotarget protein that is critical for OS cell progression.

## SUPPLEMENTARY FIGURES


