# Supplementary material for: SphK2 over-expression promotes osteosarcoma cell growth
Source: Oncotarget. 2017 Nov 6;8(62):105525–35. doi: 10.18632/oncotarget.22314 (PMC5739656; doi:10.18632/oncotarget.22314)
Supplement: Supplementary file 1 [file oncotarget-08-105525-s001.pdf]

SphK2 over-expression promotes osteosarcoma cell growth

SUPPLEMENTARY MATERIALS

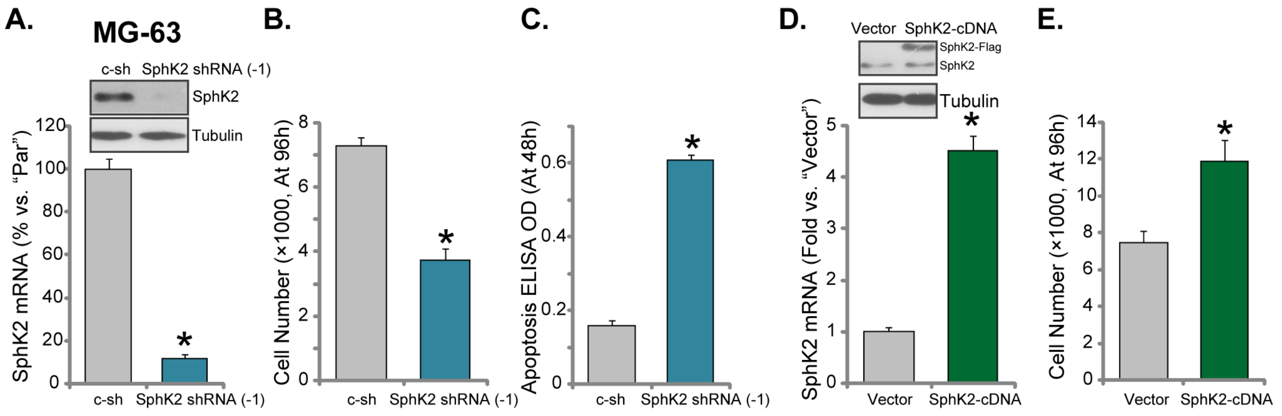

Supplementary Figure 1: mRNA and protein **(A** and **D)** expression of SphK2 in stable MG-63 cells, expressing SphK2 shRNA ("-1") or scramble non-sense control shRNA ("c-sh"), as well as SphK2 cDNA or the empty vector ("Vector", pSuper-EGFP-puro-Flag), were shown. Cells were also subjected to cell counting assay **(B** and **E)** to test cell growth; Cell apoptosis was quantified by Histone DNA apoptosis ELISA OD **(C)**. Data were shown as mean (n=5) ± standard deviation (SD). $^{*}p<0.05$ vs. "c-sh" or "Vector" cells. Experiments in this figure were repeated three times, and similar results were obtained.

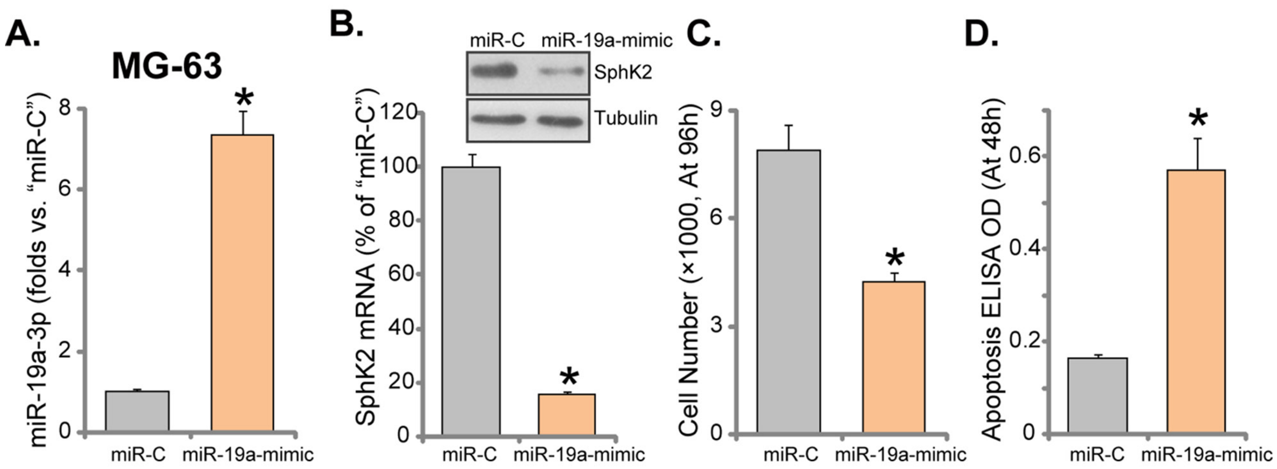

Supplementary Figure 2: Expressions of *miR-19a-3p* **(A)** and SphK2 (protein and mRNA, **B)** in MG-63 cells with miR-19a-mimic or miR-control ("miR-C") were shown; Cells were also subjected to the cell counting assay **(C)** and Histone DNA apoptosis ELISA assay **(D)**. Data were shown as mean (n=5) ± standard deviation (SD). $^{*}p<0.05$ vs. "miR-C" cells. Experiments in this figure were repeated three times, and similar results were obtained.
